# Derivation and long-term maintenance of porcine skeletal muscle progenitor cells

**DOI:** 10.1038/s41598-024-59767-0

**Published:** 2024-04-23

**Authors:** Susan O. Dan-Jumbo, Susanna E. Riley, Yennifer Cortes-Araya, William Ho, Seungmee Lee, Thomas Thrower, Cristina L. Esteves, F. Xavier Donadeu

**Affiliations:** Division of Translational Bioscience, The Roslin Institute and Royal (Dick) School of Veterinary Studies, University of Edinburgh, Midlothian, UK

**Keywords:** Porcine, Skeletal muscle, Myogenesis, Adipogenesis, Satellite cells, Biological techniques, Biotechnology, Stem cells

## Abstract

Culture of muscle cells from livestock species has typically involved laborious enzyme-based approaches that yield heterogeneous populations with limited proliferative and myogenic differentiation capacity, thus limiting their use in physiologically-meaningful studies. This study reports the use of a simple explant culture technique to derive progenitor cell populations from porcine muscle that could be maintained and differentiated long-term in culture. Fragments of semitendinosus muscle from 4 to 8 week-old piglets (n = 4) were seeded on matrigel coated culture dishes to stimulate migration of muscle-derived progenitor cells (MDPCs). Cell outgrowths appeared within a few days and were serially passaged and characterised using RT-qPCR, immunostaining and flow cytometry. MDPCs had an initial mean doubling time of 1.4 days which increased to 2.5 days by passage 14. MDPC populations displayed steady levels of the lineage-specific markers, PAX7 and MYOD, up until at least passage 2 (positive immunostaining in about 40% cells for each gene), after which the expression of myogenic markers decreased gradually. Remarkably, MDPCs were able to readily generate myotubes in culture up until passage 8. Moreover, a decrease in myogenic capacity during serial passaging was concomitant with a gradual increase in the expression of the pre-adipocyte markers, CD105 and PDGFRA, and an increase in the ability of MDPCs to differentiate into adipocytes. In conclusion, explant culture provided a simple and efficient method to harvest enriched myogenic progenitors from pig skeletal muscle which could be maintained long-term and differentiated in vitro, thus providing a suitable system for studies on porcine muscle biology and applications in the expanding field of cultured meat.

## Introduction

Skeletal muscle contains a variety of resident progenitor cell types including myogenic (predominantly derived from PAX7-expresing satellite cells) and fibro-adipogenic (a heterogeneous population within muscle), among others^1–3^. By generating the wide range of cells and tissues contained within normal skeletal muscle including myofibres, neurons, blood vessels, adipose and connective tissue, these diverse progenitor populations play key roles in tissue development and regeneration in response to disease, injury or exercise^4,5^. The ability to selectively grow and manipulate myogenic progenitors in vitro has provided considerable insight onto skeletal muscle development and function both in health and disease, as well as allowing unprecedented advances into novel tissue engineering approaches for muscle regeneration^6^. Two key challenges to understanding the biology of myogenic progenitors have been (1) the limited abundance of satellite cells in muscle tissue samples, and complexities associated with robust isolation of pure populations of such cells, and (2) their short lifespan in vitro due to the propensity of satellite cells, once they are taken from their natural niche, to rapidly differentiate irreversibly into committed progenitors termed myoblasts which, upon an inductive environment will readily fuse into myotubes.

Apart from human biomedicine, the development of robust in vitro models of skeletal muscle has enormous potential in the field of meat production, both for the study of muscle biology in livestock and in the newer and highly expanding area of cellular agriculture. In this regard, pigs are the most consumed meat-producing species globally, in addition to a growing interest in their use as biomedical models^7^. Yet, efforts to establish and adequately characterise in vitro models of skeletal muscle in pig have been limited^8,9^.

Harvesting myogenic progenitors from tissue samples in model species has typically involved enzymatic digestion of skeletal muscle fragments together with size-based selection by filtration to obtain a mononuclear cell population^10,11^. These are sometimes purified further using pre-plating, density gradient centrifugation, fluorescence-activated cell sorting (FACS) or magnetic activated cell sorting (MACS). The latter two approaches can yield relatively pure fractions of myogenic progenitors^12–14^, however they have the major drawback that surface markers are not universally available, limiting their use in species other than rodents and humans, in addition to imposing mechanical stress which may impair cell survival and proliferation^15–17^. Moreover, both FACS and MACS can be relatively laborious and the purity of cells obtained is variable often resulting in an overgrowth by fibroblasts within a week of culture^18–20^.

An alternative to enzymatic digestion and purification has been the explanting of muscle tissue fragments to allow progenitor cell populations to migrate out during incubation. This method, which indeed preceded the use of enzymes for this purpose, was originally used to isolate and culture single fibres from rat muscle with the aim to create an in vitro system to maintain satellite cells in their the natural niche^21^. The method has since gained popularity and has been adapted to different species and conditions^22–28^. An important advantage of the explant compared to the above methods is that it minimises the trauma to myogenic progenitors during harvesting. In addition, the action of cutting up muscle samples into smaller pieces for explanting may be itself akin to muscle trauma thereby triggering satellite cell activation, migration, and proliferation by promoting the release of soluble factors as well as growth factors that naturally supports the survival and proliferation of the emerging progenitor populations^29,30^, thus in principle making explant culture a good mimicry of the in vivo environment.

Some of the above approaches have been used to enrich progenitor populations from enzymatically digested muscle in the pig^8,9,31–34^. However, the identity and long term multi lineage differentiation capacity of those cell populations were not adequately characterized. Moreover, only one study reported using the explant method, and while the cells obtained showed long term expansion, they were akin to mesenchymal stem cells (MSCs), both in terms of immunophenotype and differentiation abilities, but did not express muscle stem cell markers and failed to form myotubes in vitro except when co-cultured with murine C2C12 myoblasts, indicating that they were not myogenic in nature^35^.

The aim of this study was to develop robust methodology for enrichment and long-term expansion of myogenic progenitors from pig skeletal muscle using explant cultures. The study provides for the first time detailed characterisation of muscle-derived progenitor cell (MDPC) populations thus obtained (the term MDPC including both myogenic and adipogenic progenitor cells), in terms of their proliferative capacity, expression of key lineage markers, and ability to differentiate into different cell lineages during serial passaging in vitro.

## Results

### Derivation of MDPC cultures

**Figure 1:**
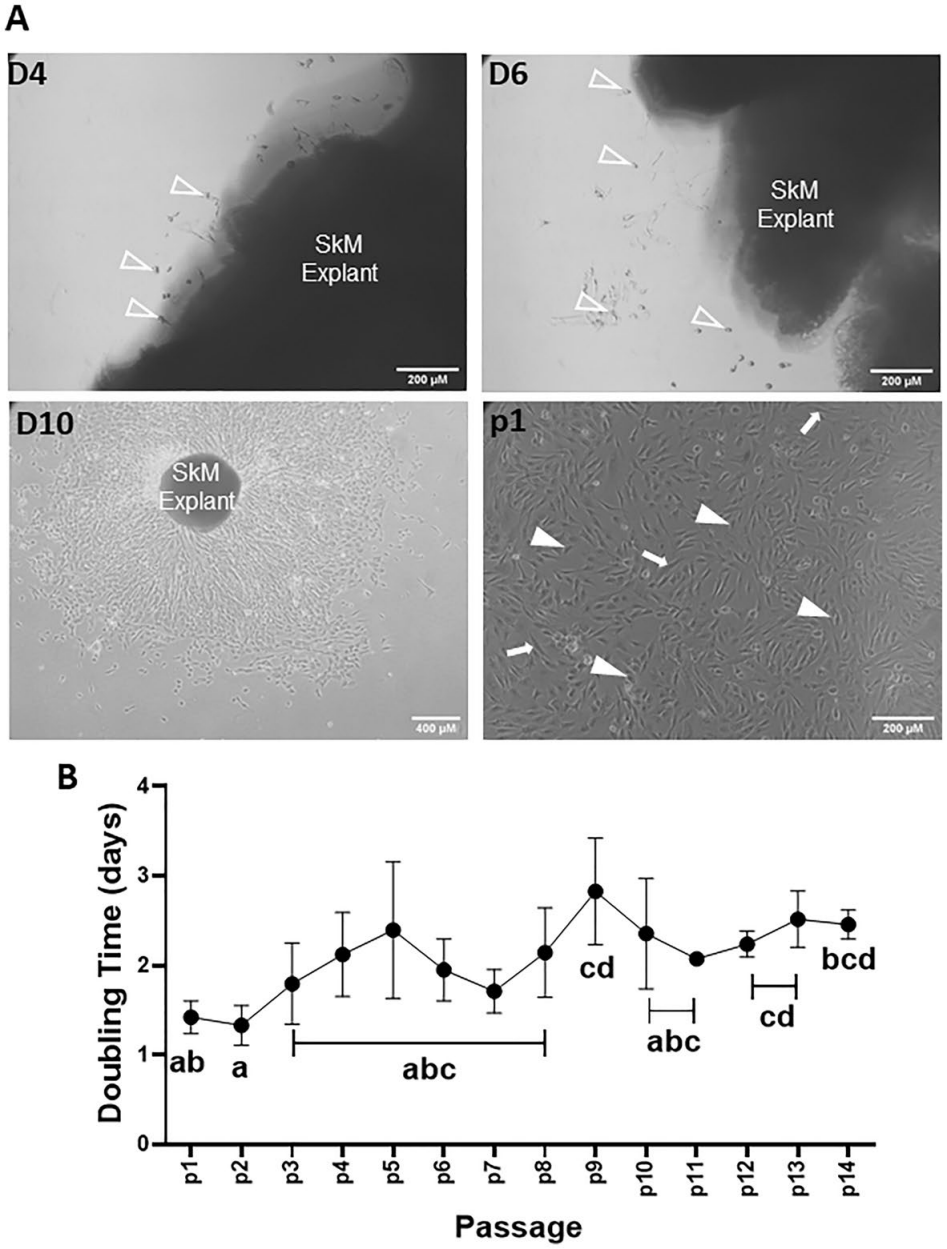
Derivation of porcine MDPC cultures. (**A**) Representative phase contrast images of skeletal muscle explants at 4, 6 and 10 days (D4, D6, D10) after seeding in matrigel-coated plates, and of MDPC cultures after one passage (p1). Structures identified by arrows and arrowheads are referred to in the Results. (**B**) MDPC doubling times (mean ± S.E.M) over passages 1–14 (P < 0.0001, n = 4 animals). Means with different subscripts (abcd) are significantly different (P < 0.05).

Cell egression from muscle fragments was detected within 4 days of explant culture. On Days 4 and 6 after explant, most outgrowing cells were small and compact (Fig. 1A, white open arrowhead). Outgrows were allowed to expand for about 2 weeks after which they were passaged. At that point, most cells had become spindled-shaped (Fig. 1A, p1, white closed arrowhead), consistent with a transition, within myogenic populations, from activated satellite cells to myoblasts. A few cells appeared flat and elongated and were most likely fibroblasts (Fig. 1A, p1, white arrows). Cells were then serially passaged at low density (5000 cells/cm^2^) in proliferation media for up to 50 days (14 passages), during which their doubling time increased from 1.42 ± 0.16 days in passage 1 (p1) to 2.41 ± 0.02 days in p14 (Fig. 1B) indicating a reduction in growth rate with passaging.

### Expression of lineage-specific and cell surface markers by porcine MDPCs

Changes in the expression of different myogenic and mesenchymal progenitor markers were analysed by RT-qPCR, immunofluorescence and/or flow cytometry following serial passaging of MDPCs.

#### Myogenic progenitor markers

**Figure 2:**
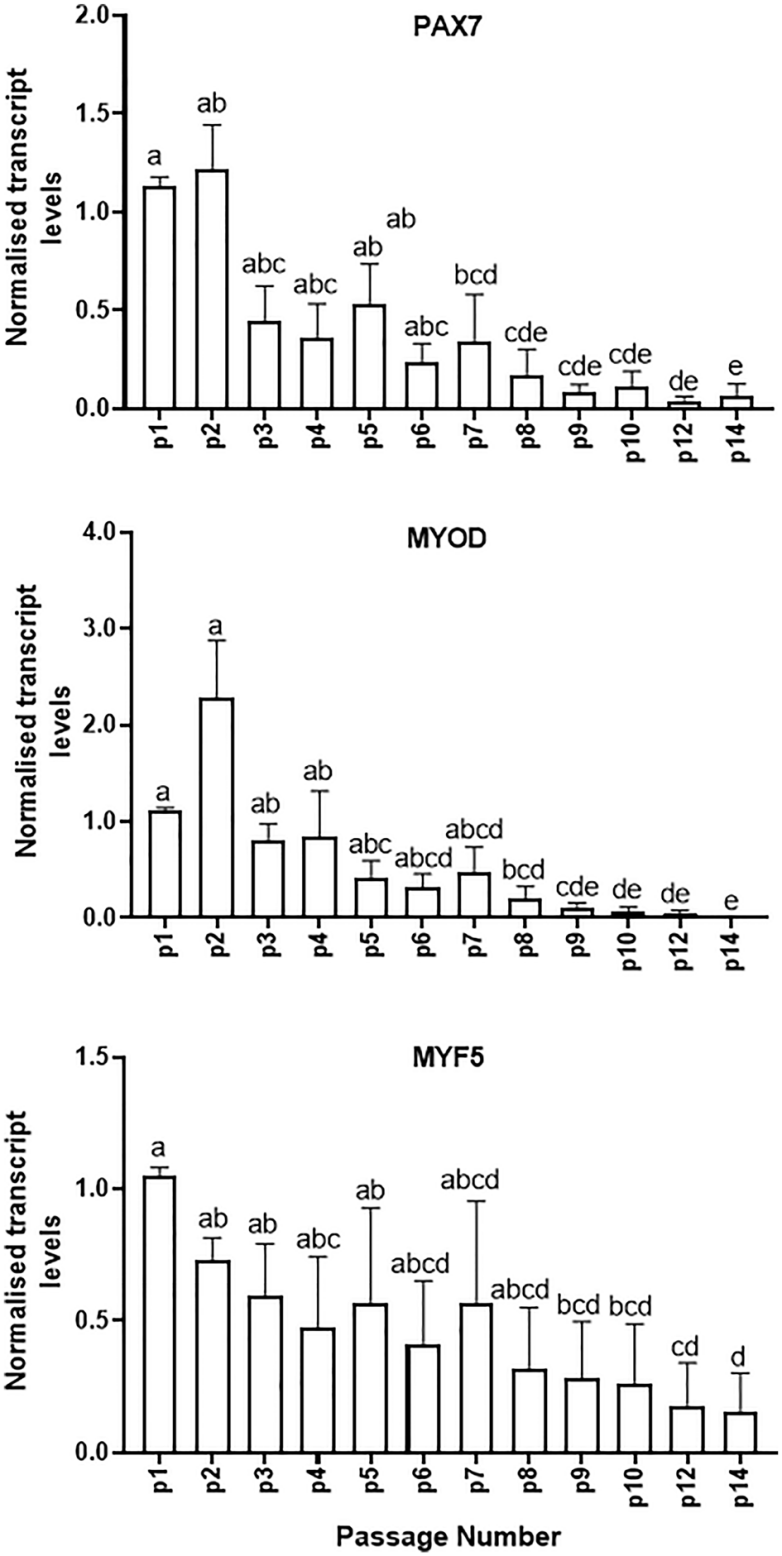
Changes in levels of muscle-specific transcripts in MDPCs. Relative mRNA levels (mean ± S.E.M) of *PAX7, MYOD* and *MYF5* in MDPCs at different passages (p). For each animal, expression values for each passage were normalised to p1 values. For all transcripts, the effect of Passage number was significant (P < 0.0001). Different superscripts (abcde) indicate significant differences between means (P < 0.05). n = 4 animals.

**Figure 3:**
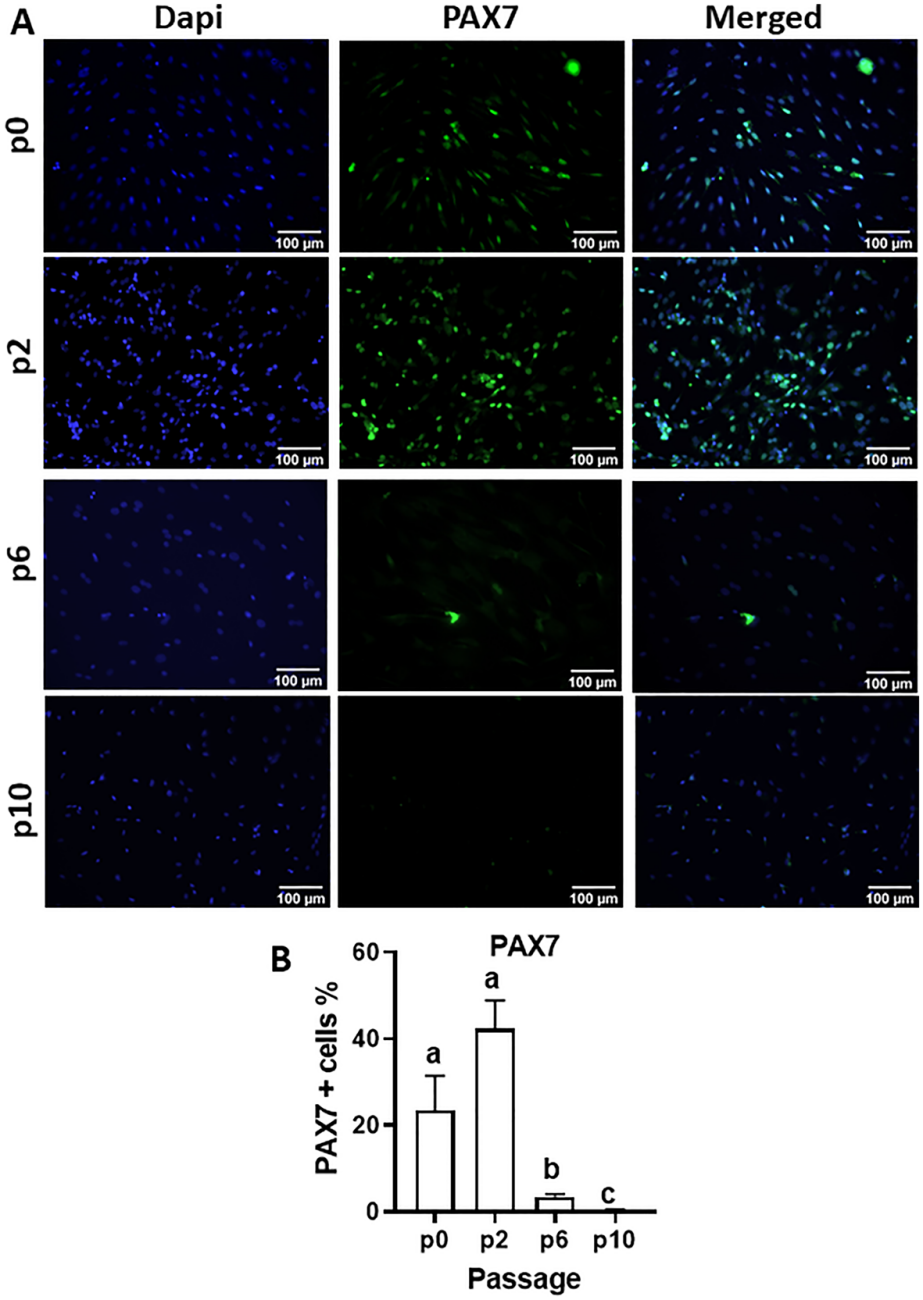
Changes in PAX7-positive cells during MDPC culture. (**A**) Representative images of porcine MDPCs at p0 (i.e. day 7 of muscle explant, before passaging) and at passages 2, 6 and 10, that were immunostained with PAX7 (green); blue shows DAPI staining. (**B**) Percentages of cells (Mean ± S.E.M) that stained positive for PAX7 at each passage (P < 0.0001; n = 3 animals). Means with different superscripts (abc) are different (P < 0.05).

**Figure 4:**
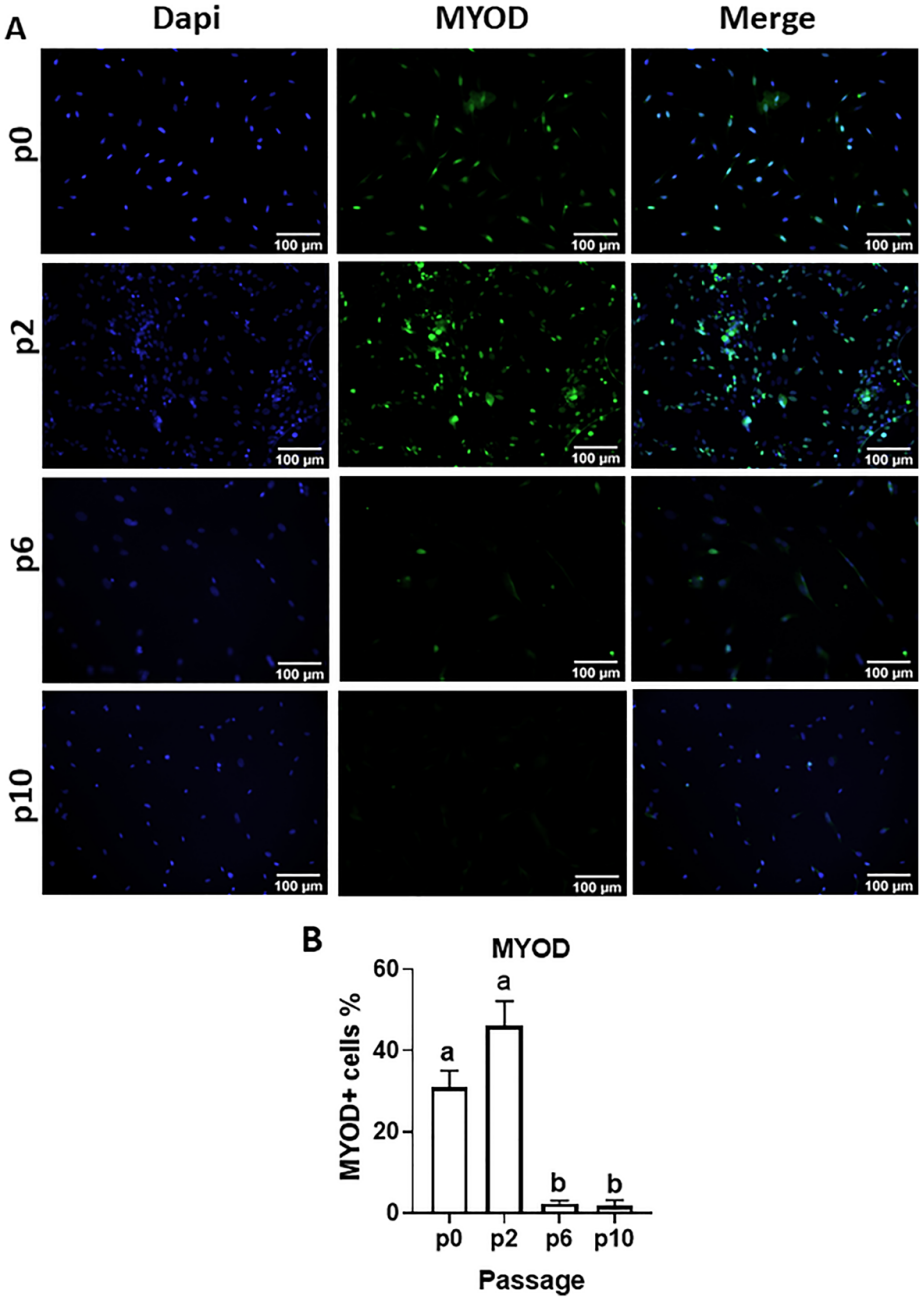
Changes in MYOD-positive cells during MDPC culture. (**A**) Representative images of porcine MDPCs at p0 (i.e. day 7 of muscle explant and before passaging) and at passages 2, 6 and 10, that were immunostained with MYOD (green); blue shows DAPI staining. (**B**) Percentages of cells (Mean ± S.E.M) that stained positive for MYOD at each passage (P < 0.01; n = 3 animals). Means with different superscripts (ab) are different. (P < 0.05).

Mean mRNA levels of the lineage-specific transcription factors, *PAX7, MYOD and MYF5*, were relatively stable in MDPCs up to p2, and decreased progressively thereafter until p14 (Fig. 2). Consistent with this, the percentages of cells immunostained for PAX7 (Fig. 3) and MYOD (Fig. 4) decreased dramatically at p6 and p10 relative to p2 (12- and 128-fold reduction, respectively, for PAX7, and 19- and 27-fold reduction for MYOD). Immunostaining was also performed on cell outgrowths at p0, i.e. 7 days after explanting and before cells were passaged (Figs. 3A and 4A, top lanes); for both PAX7 and MYOD, percentages of positive cells were not significantly different (P > 0.1) between p0 and p2 (Figs. 3B and 4B).

**Figure 5:**
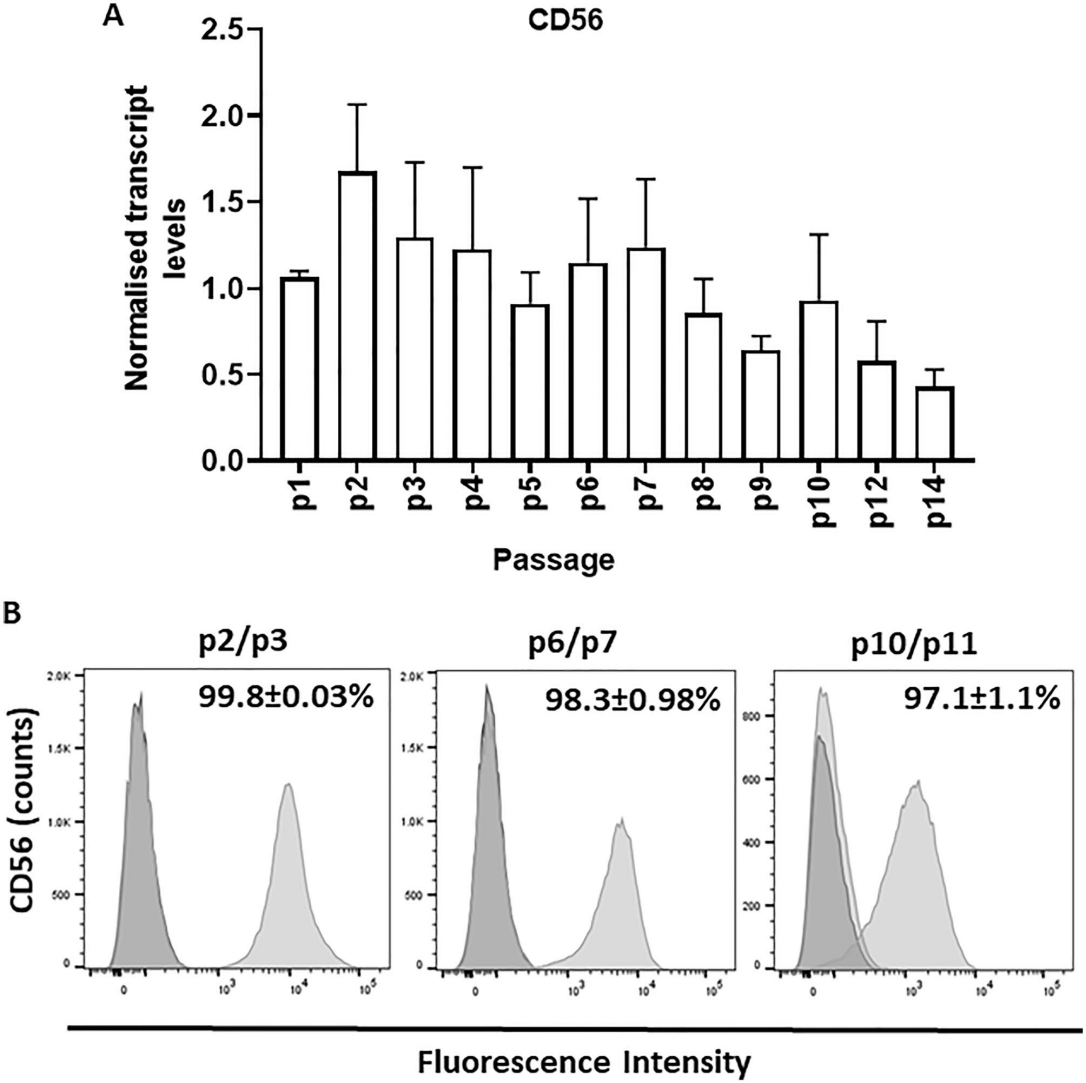
Changes in CD56 expression in MDPCs. (**A**) Relative mRNA levels of *CD56* in MDPCs (mean ± S.E.M) quantified by qRT-PCR across different passages in culture. For each animal, expression values for each passage were normalised to p1 values. (**B**) Representative flow cytometry histograms showing percentages of positive CD56-positive MDPCs (right light grey curve). Signal from the corresponding isotype controls is shown (left dark grey curve). Percentages (mean ± S.E.M) of CD56-positive cells are indicated (n = 3–4 animals).

In addition, mRNA levels of *NCAM*, also known as *CD56*, a cell surface marker associated with myogenic progenitors, did not change significantly with serial passaging (Fig. 5A). In addition, flow cytometry analyses revealed a distinct peak for CD56 positive cells which, at all passages analysed, included a majority of cells in culture (Fig. 5B).

#### Mesenchymal progenitor markers

**Figure 6:**
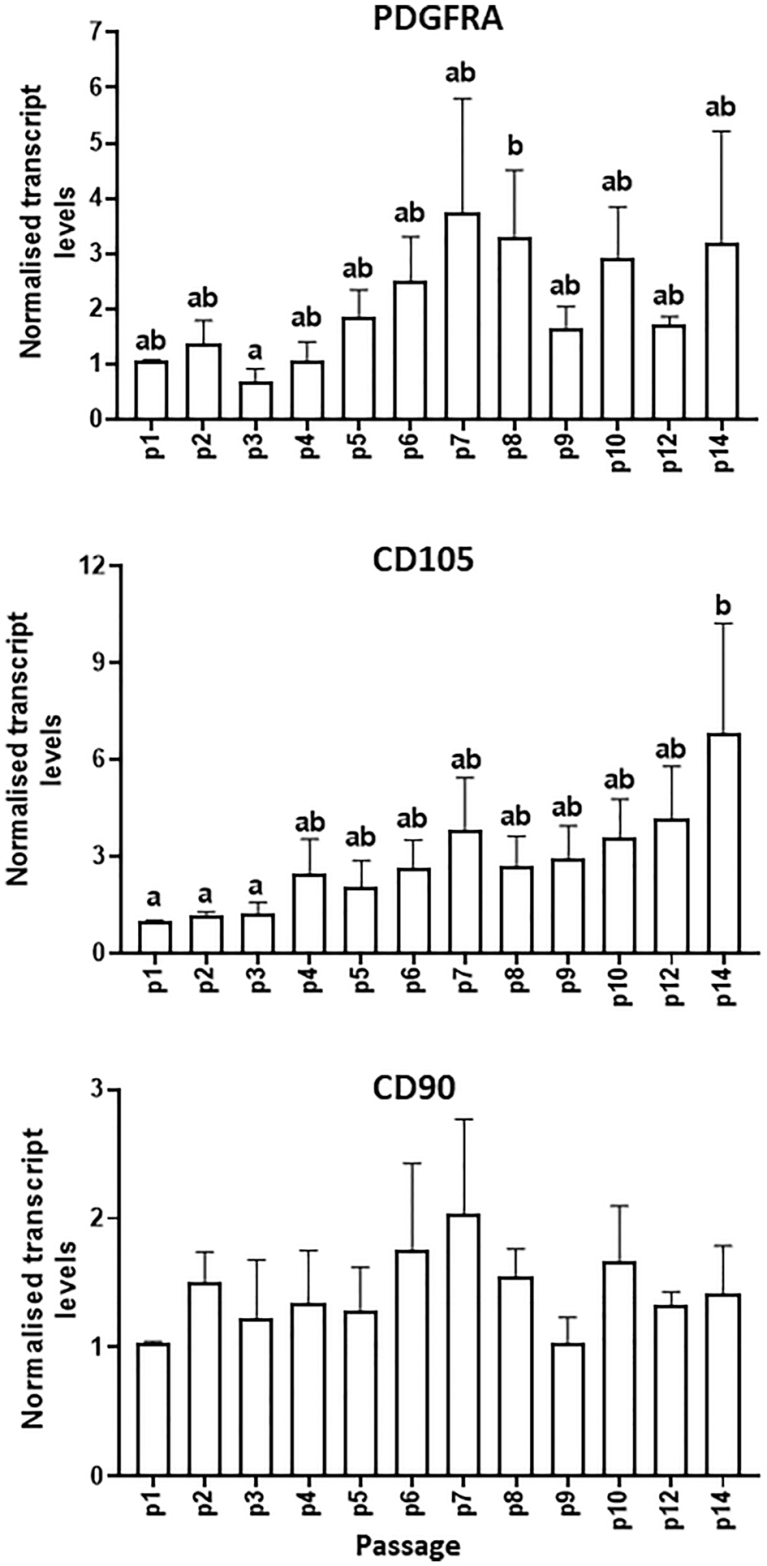
Changes in levels of mesenchymal progenitor markers in MDPCs. Relative transcript levels (mean ± S.E.M) of *PDGFRA, CD105* and *CD90* in MDPCs at different passages. For each animal, expression values for each passage were normalised to p1 values. There was an effect of Passage number for *PDGFRA* (P = 0.036) and *CD105* (P = 0.001). For any given marker, means with different superscripts (ab) are different (P < 0.05). n = 4 animals.

**Figure 7:**
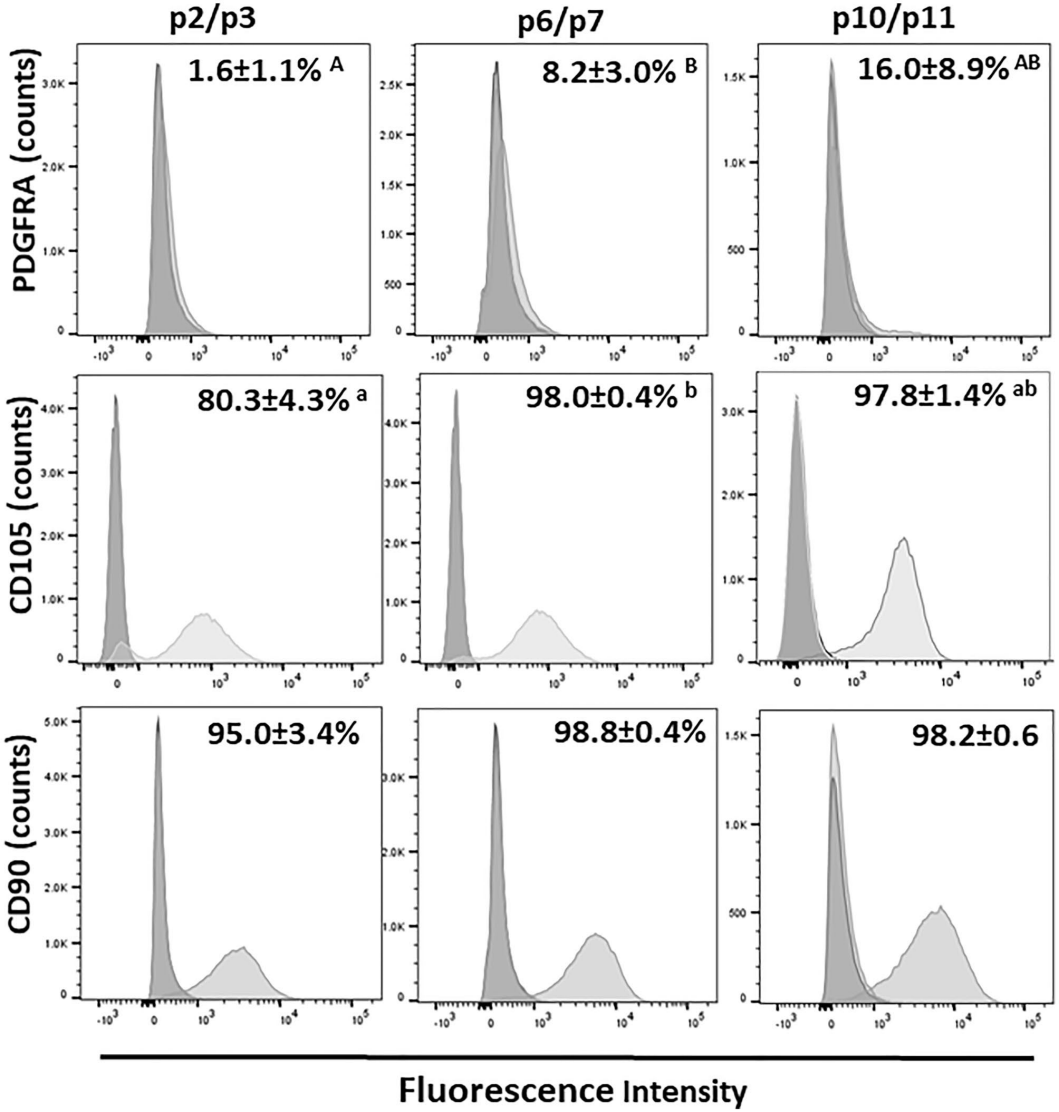
Changes in the percentages of cells positive for different mesenchymal markers as determined by flow cytometry. Representative flow cytometry histograms showing percentages of MDPCs positive (right light grey curve) for PDGFRA, CD105 and CD90 during serial passaging. Signal from the corresponding isotype controls is shown in dark grey displayed on the left, and percentage of positive cells (mean ± S.E.M) is also shown. There was a significant effect of Passage number for CD105 (P = 0.036). For a given marker, percentage values with different superscripts were significantly different (ab; P < 0.05) or approached significance (AB; P < 0.1). n = 3 animals.

Levels of the cell surface markers, PDGFRA (also known as CD140a), CD105 and CD90 in MDPCs were quantified by both RT-PCR (Fig. 6) and flow cytometry (Fig. 7). Results showed an increase in mean *PDGFRA* transcript levels with serial passaging resulting in significantly higher values at P8 relative to P3. Likewise, mean levels of *CD105* mRNA increased progressively during serial passaging, whereas *CD90* remained unchanged (P > 0.1). Moreover, flow cytometry analyses showed that the percentage of PDGFRA-positive cells was, on average, very low (< 2%) at p2/p3, and increased, although not significantly, with serial passaging (Fig. 7; p2/p3 vs. p6/p7, P = 0.08). Moreover, mean percentages of CD105-positive and CD90-positive cells were very high (> 80%) already by p2/p3; consistent with RT-qPCR data, the percentage of CD105-positive cells increased further during serial passaging (Fig. 7). In addition to the above MSC markers, the levels of the hematopoietic marker, CD45, were also analysed, however CD45 was not detected by flow cytometry (data no shown), suggesting the absence of hematopoietic cells in MDPC cultures. Finally, mean fluorescence intensity values across passages did not change (P > 0.1) for any of the markers analysed.

### Differentiation capacity of porcine MDPCs

Finally, the ability of MDPCs to differentiate into the two key skeletal muscle lineages, myogenic and adipogenic, as well as other mesenchymal lineages, namely bone and cartilage, was assessed.

#### Myogenic differentiation

**Figure 8:**
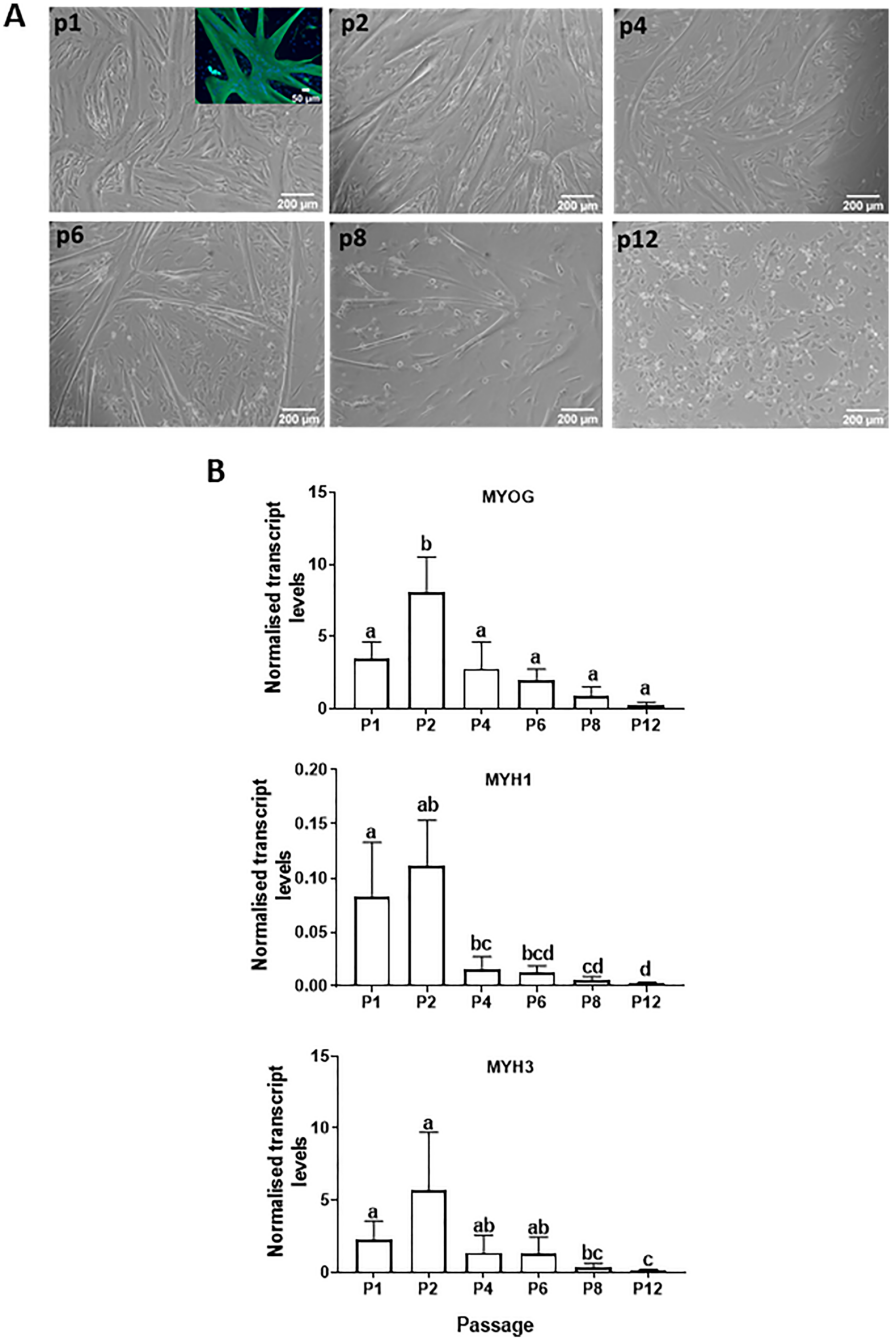
Myogenic capacity of MDPCs. (**A**) Bright field images showing myotubes resulting from 3-day differentiation of MDPCs at selected passages (p). Insert shows a representative image of myotubes immunostained for myosin heavy chain (MYHC shown in green, Dapi in blue). (**B**) Relative levels (mean ± S.E.M) of *MYOG, MYH1*, and *MYH3* transcripts in MDPCs differentiated for 3 days at different passages. There were significant effects of Passage number for all three transcripts (P < 0.001). Values are displayed as fold change expression relative to values on Day 0. n = 3 animals. Means with different superscripts (abcd) are different (P < 0.05).

The ability of MDPCs to form myotubes in vitro was assessed at selected time-points from p1 to p12 (Fig. 8). When placed under differentiation conditions, porcine MDPCs produced visible multinucleated myotubes at all passages analysed except p12, however, differentiation efficiency decreased after p2 as indicated by thinner and fewer myotubes (Fig. 8A). This was confirmed by quantification of the myogenic transcript, *MYOG*, together with the developmental myosin heavy chain isoforms *MYH1* and *MYH3*, the levels of all of which decreased accordingly with passage number (Fig. 8B).

#### Adipogenic differentiation

**Figure 9:**
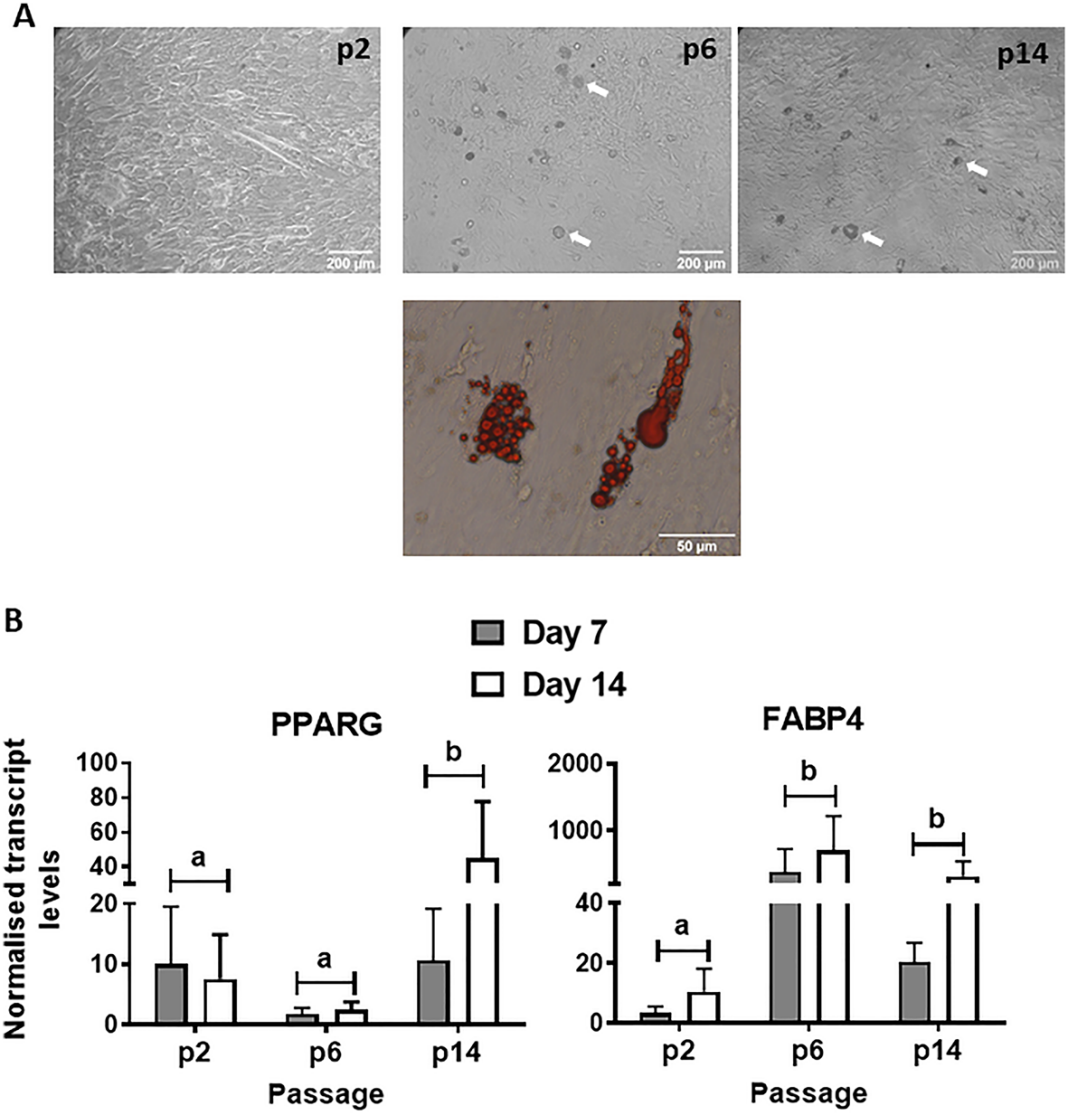
Adipogenic capacity of MDPCs. (**A**, upper panels) Representative bright-field images of MDPCs at different passages that were maintained in adipogenic media for 14 days. Adipocytes containing lipid droplets are shown by white arrows. (**A**, lower panel) Image showing oil red O staining of intracellular lipid droplets. (**B**) Relative mRNA levels (mean ± S.E.M) of *PPARG* and *FABP4* in MDPCs at p2, p6 and p14 after 7 and 14 days in adipogenic induction media. There were significant effects of Passage number for both PPARG (P = 0.001) and FABP4 (P = 0.049). Values are displayed as fold change expression relative to the Day 0 values. n = 4 animals. Means with different superscripts (ab) are significantly different (P < 0.01).

Adipogenic capacity was determined in cells at p2, 6 and 14 (Fig. 9). After 14 days in adipogenic media, p2 cells remained elongated and did not show lipid accumulation (Fig. 9A, upper panel). However, for both p6 an p14, some cells became rounded and displayed visible lipid droplets upon adipogenic induction (Fig. 9A, upper panel), as confirmed by oil-red-oil staining (Fig. 9A, lower panel). Moreover, qPCR analyses of the transcriptional activator, *PPARG*, and the fatty acid carrier protein, *FABP4*, at 7 and 14 days after adipogenic induction confirmed an increase in adipogenic activity in MDPC cultures at p6 and p14 compared to p2 (Fig. 9B). Taken together, these findings indicate that porcine MDPCs gained adipogenic potential with serial passaging concomitant with the loss of myogenic capacity.

#### Osteogenic and chondrogenic differentiation

To further investigate their plasticity, the ability of MDCPs to differentiate into two additional mesenchymal lineages, namely, bone and cartilage, was assessed. Seeding cells at p3 in appropriate differentiation conditions (see “Methods” section) resulted in changes consistent with osteocyte differentiation, as evidenced by Alizarin Red staining (Supplementary Fig. 1), however, MDPCs failed to produce clear chondrogenic pellets.

#### Clonal differentiation

Whether myocytes and adipocytes originate from distinct or the same progenitor populations in muscle is still the subject of controversy. To investigate this in the pig, early passage MDPCs were seeded as single cells and expanded in 96 well plates followed, once they reached confluence, by induction of differentiation separately into each of the two lineages. A total of 70 clones from two different animals (35 clones from each) were analyzed. In total, 18 out of the 70 clones (26%) were able to produced adipocytes, as evidence by Oil Red O staining (Supplementary Fig. 2), whereas MYHC-stained myotubes could not be identified from any clone. Together with a lack of temporal association between myogenesis and adipogenesis during MDPC culture (see above), these results are consistent with separate progenitor populations largely giving rise to the two lineages in pig MDPCs.

## Discussion

The availability of reliable procedures for robust expansion and maintenance in culture of enriched myogenic progenitor populations from livestock species would prove highly valuable for both basic and applied studies to improve the efficiency of animal meat production, including the emerging area of cell-based foods, as well as for the use of large animals as models of muscle disease. The present study provides a step forward towards achieving that by reporting for the first time in the pig the successful derivation and characterisation of a tissue explant-derived culture system allowing long-term maintenance of myogenic progenitors contained within MDPCs, and which provides several advantages over the classic use of enzyme-based cell isolation methods in this species. The explant method, more commonly used in traditional model species, takes advantage of the natural ability of satellite cells to become activated upon injury (induced by mechanical mincing in this case), migrate and proliferate rapidly to repair damaged tissue^22,26,27,36^.

Pig muscle explants gave rise to fast-growing cell outgrowths which, 7 days after initiation of explant culture, contained a proportion of myogenic progenitors ranging between 12 and 39%, based on staining for the lineage-specific markers, PAX7 and MYOD (Figs. 3 and 4). These values are comparable to those obtained from human muscle explants (10–50% Pax + cells)^28^ but lower than those reported using mouse muscle (60–90% myogenic progenitors)^37^. Moreover, the proportion of myogenic cells, as well as their ability to robustly generate myotubes, were maintained at relatively stable levels in porcine explant-derived cultures until at least passage 2. In addition, MDPCs maintained their capacity to generate myotubes until at least passage 8, corresponding to about 42 days after the beginning of explant culture. Loss of stemness during in vitro culture is a well-known feature of myogenic progenitors from several species^38,39^ including pig^8^. A study in which porcine satellite cells were sorted based on CD56 and CD29 expression^8^ reported a 30-fold decrease in PAX7 expression after 96-h in culture, with a continued more gradual decrease in both PAX7 expression and differentiation capacity during sequential passaging up to passage 10, when myotube formation became undetectable. The present study achieved similar results using a much simpler, less laborious approach to obtain myogenic progenitors. Previously reported strategies such as addition of p38 kinase inhibitors^40^ or muscle-secreted cytokines^38^ may prove beneficial in further extending the myogenic potential of porcine explant-derived MDPCs during culture.

To our knowledge, only one previous study^35^ had reported the use of explant culture as a source of myogenic cells in the pig, however, those cells did not express PAX7 or MYOD and were not able to undergo myogenic differentiation unless co-cultured with C2C12 cells, although they were capable of undergoing adipogenesis and osteogenesis. The use of gelatin rather than a laminin-rich substrate such as matrigel for cell expansion may have accounted for the inability to obtain myogenic-rich cell populations in that study. Matrigel, such as used in our study, has been shown to distinctly support the migration and proliferation of mice and human myoblasts while maintaining their myogenic capacity during prolonged culture in vitro^41,42^.

The cell adhesion protein, CD56, localises to satellite cells in skeletal muscle and has been widely used to selectively harvest tissue myogenic progenitors, including in the pig^8^. The present study sought to determine whether, similar to PAX7 and MYOD, CD56 could be use as marker of myogenic potential of pig MDPCs. Of note, almost all MDPCs were CD56-positive and remained as such throughout culture, despite a clear and dramatic reduction in the abundance of myogenic progenitors with sequential passaging (Fig. 5). Ding et al. ^8^ found that CD56 levels decreased in porcine satellite cells during culture, although to a lesser extent than PAX7 levels or myogenic capacity did. Moreover, different cell types, including MSCs^43^ have been shown to express CD56 in addition to muscle stem cells, whereas recent work showed that CD56-negative cells in porcine muscle were able to generate myotubes in culture^44^. Taken together, these data suggests that, although suitable for identifying myogenic progenitors in native skeletal muscle, CD56 does not provide a specific lineage marker in porcine MDPCs in culture.

The present results show that porcine muscle explant-derived cultures harboured a dynamic population of multipotent adipose progenitors. This was indicated by (1) positive staining of MDPCs for the mesenchymal progenitor markers, PDGFRA, CD105 and CD90, together with multilineage differentiation capacity (fat, bone), and (2) the upregulation of PDGFRA and CD105 together with an increase in adipogenic capacity during extended culture. In support of this conclusion, PDGFRA is a canonical marker of adipogenic progenitors in skeletal muscle^5,45–47^, whereas CD105, but not CD90, marked multipotent, adipogenesis-competent precursor cell populations in muscle^48^. Our findings suggest that, in contrast to the relatively rapid loss of myogenic progenitors during passaging, adipogenic precursors in explant cultures were able to maintain the capacity for self-renewal resulting in an overall increase in adipogenic capacity up until at least passage 14. This is consistent with the ability of tissue derived porcine MSCs to robustly expand and differentiate into adipocytes after extended passaging in culture^49–52^.

There is significant evidence, both in vitro and in vivo, of a dynamic crosstalk between myogenic precursors and fibro-adipogenic precursors (FAPs) in skeletal muscle^4,5,53–55^, including inhibition of FAP adipogenesis by muscle-secreted products^56–59^ or by the presence of myotubes in culture^45^. Those observations may to an extent explain the temporal association observed in this study between increased adipogenic capacity and a loss in myogenic capacity of MDPCs. Moreover, although not convincingly demonstrated to this date, the presence of bipotent precursors able to switch from a myogenic to an adipogenic fate in response to changing conditions upon extended passaging cannot be discarded in explant cultures^60–62^. Yet, results of clonal cell analyses in the present study failed to demonstrate the presence of bipotent progenitors in porcine MDPCs. A caveat to this conclusion was the rapid loss of myogenic capacity during clonal expansion of MDPCs, in light of which, provided suitable lineage-specific antibodies for pig become available in the future, lineage tracing studies using specific myogenic and adipogenic markers may be needed to more definitely rule out the presence of bipotent progenitors.

## Conclusion

This study reports for the first time the robust isolation and expansion in vitro of both myogenic and adipogenic precursors from pig skeletal muscle using explant culture, and provides detailed characterisation of their dynamics during extended passaging in culture. It is shown that, under the specified conditions, a high proportion of differentiation-competent myogenic progenitors can be maintained for at least two passages, and that complete loss of myogenic precursors occurs only after passage 8. Moreover, a progressive decrease in myogenic cells during serial passaging was temporally associated with a gradual increase in adipogenic precursors up until at least passage 14. In conclusion, this study provides a new, relatively simple and convenient system to study porcine muscle progenitor cell dynamics in culture.

## Methods

### Isolation and culture of MDPCs

All animal procedures were performed with approval from The Roslin Institute (University of Edinburgh) Animal Welfare and Ethical Review Board and following the UK Animals (Scientific Procedures) Act, 1986. All experiments were performed in accordance with relevant guidelines and regulations. All procedures were performed according to ARRIVE guidelines.

Four Landrace piglets (4–8 weeks old) were euthanized by intravenous injection of sodium pentobarbitone 20% w/v (Henry Schein Animal Health, Dumfries, UK). Once death was confirmed, the hind limb was dissected aseptically and samples of semitendinosus muscle (10 g) were immediately transferred to phosphate buffered saline (PBS) supplemented with 2.5 µg/ml Amphotericin B (Life Technologies, Carlsbad, CA, USA) and 1% penicillin–streptomycin (PS; Life Technologies), and transported on ice to the laboratory. Explant cultures were then set-up following a protocol adapted from Shahini et al. ^26^. First, pieces of muscle tissue were washed in three changes of the above cold PBS solution, carefully dissected to remove fat and connective tissue, and finally minced into small tissue fragments that were washed again in 2 changes of the same solution, prior to plating on matrigel (BD Biosciences, Franklin Lake, NJ, USA) in 6 well tissue culture plates with fresh proliferation media consisting of Hams F10 nutrient mix (Life Technologies, Carlsbad, CA, USA) supplemented with 20% Foetal Bovine Serum (FBS, Life Technologies), 1% PS, 2.5 μg/ml amphotericin B and 5 ng/ml bFGF (PeproTech, London, UK), at 39 °C with 5% CO_2_. Cells were observed every other day under a brightfield microscope and fresh media was added as needed. After a maximum of 14 days in culture, tissue fragments were removed, and cell monolayers washed with PBS prior to detaching the cells with 0.25% Trypsin–EDTA (Fisher Scientific UK), after which they were either frozen in 5% DMSO:95% FBS or further grown as MDPCs. These were expanded by re-plating trypsinised cells on tissue culture flasks coated with 0.1% gelatin (Merck Life Science, UK) at a density of 5000 cells/cm^2^ in proliferation media. Cells were maintained at a density of < 70% confluence and counted using Trypan Blue solution (Sigma Aldrich, St Louis, MO, USA) at each passage. Doubling times were calculated using the formula:

$$Doubling\;Time = \frac{Time\;in\;culture \times \log (2)}{{\log (Final\;number) - \log (Initial\;number)}}$$

where Initial number = number of cells seeded, and Final number = number cells at harvesting.

### Clonal MDPC derivation and analyses

MDPCs were dissociated into single cells, washed in PBS, and resuspended in FACS buffer (1% BSA in PBS) at a final concentration of 1 × 10^6^ cells/ml before single cell sorting using the BD Fortessa X20 into 96 well plates coated with 0.1% gelatine. Sorted cells were incubated untouched for 7 days in 60% proliferation media and 40% MPDC conditioned media. The latter was obtained from 70% confluent MPDCs and centrifuged at 600 × g for 5 min and filtered through a 0.22 µm filter to remove cell debris before use. Media was changed every 2–3 days until clonal populations reached approximately 70–90% confluency. Cells were then split into two fresh 96 well plates using TrypLE express (Gibco) and incubated in proliferation media before induction of either myogenic or adipogenic differentiation followed by staining with Myosin Heavy Chain or Oil Red O, as described below.

### Flow cytometry

MDPCs were dissociated into single cells, washed in PBS and re-suspended in FACS buffer (1% BSA in PBS) to a final dilution of 0.5 × 10^6^ cells/ml. Cells were then incubated with either 10% mouse serum (Sigma Aldrich) or 10% goat serum (Abcam, Cambridge, UK) in PBS at 4 °C for 30 min, followed by 1 h at 4°°C with antibodies as shown Table 1. Cells were then washed 3 × with FACS buffer and unconjugated Anti-CD105 was further incubated with APC labelled secondary antibody for 30 min. Isotype controls shown in Table 1 were used at same concentrations as their respective primary antibodies and analysed in parallel. To determine cell viability the samples were further incubated with Zombie Aqua viability dye (1000 × dilution, BioLegend, London, UK) for 15 min at room temperature in the dark and washed once in FACS buffer prior to analysis. Flow analysis was performed on BD Fortessa X20, and data analysed with FACSDiva software (BD Biosciences, San Jose, CA, USA) or FlowJo (LLC, Ashland, OR, USA).

**Table 1:** List of Antibodies and isotype controls used for flow cytometry.

Antibody	Provider	Catalogue number	Dil./conc.
Pax7	R&D laboratories	MAB1675	5 μg/ml
MYOD	Agilent Technologies	M351201	20 μg/ml
MYHC	R&D laboratories	MAB4470	5 μg/ml
CD45-FITC	AbD Serotec-BioRad	MCA1222F	1:20
CD56-BV421	BD Biosciences	562751	1:50
CD90-BV605	BD Pharmingen	747750	1:100
CD140a-PE	BioLegend	323505	1:25
CD105	Abcam	ab69772	1:40
Anti-mouse IgG2a-APC	BioLegend	407110	1:200
Isotype controls			
Mouse IgG2a, k	Abcam	ab18415	1:40
Mouse IgG1 AF647	AbD Serotec-BioRad	MCA928A647	1:50
Mouse IgG1, κ AF700	BioLegend	400144	1:25
Mouse IgG2b, k BV421	BD Biosciences	562748	1:50
Mouse IgG1, κ BV605	BioLegend	562652	1:100
Mouse IgG2a, k FITC	AbD Serotec-Biorad	MCA928F	1:20
Mouse IgG1 RPE	BD Pharmingen	MCA928	1:13

### RT-qPCR

Cell samples were collected in Trizol (Invitrogen, Carlsbad, CA, USA) and RNA extracted following manufacturer’s instructions. Total RNA (0.5 µg) was reverse transcribed (RT) using SuperScript III (Invitrogen) and random primers (Promega, Madison, WI, USA) in the presence of RNasin Plus Rnase Inhibitor (Promega), with heating to 25 °C for 5 min, 50 °C for 1 h and 70 °C for 15 min in a thermocycler. RT-qPCR was performed using Sensi-FAST SYBR Lo-ROX Kit (Bioline, London UK) according to manufacturer’s instructions, on a Strategene Mx3005P machine (Agilent, La Jolla, CA. USA). Included with each analysis was No RT control, No DNA template control and a standard curve generated from four-fold serially diluted samples obtained from a pool of all test samples. All samples and controls were run in duplicate, and copy numbers were calculated relative to the standard curve using MxPro software. Gene expression values were normalized to the average value of the reference genes, 18s and RPL4, for that sample. In addition, normalised values for each cell passage were expressed as fold-change relative to one of the triplicate values at passage 0 or passage 1 (which was taken as having a value of 1; see Figure legends for details) before statistical analyses. Primers used for gene analysis are listed in Table 2.

**Table 2:** Details of primers used for quantitative real time qRT‐PCR.

Target	Forward primer sequence 5′-3′	Reverse primer sequence 5′-3′
18s	GCTGGCACCAGACTTG	GGGGAATCAGGGTTCG
CD56	ACCTGGTCAAATACCGAGCG	TCCTGAACACGAAGTGAGCC
CD90	GACTGCCGCCATGAGAATAC	GGTAGTGAAGCCTGATAAGTAGAG
CD105	ATACAAAGGGCTCCATCATC	TGAGTGTGAGACTTCCATTC
FABP4	AACCCAACCTGATCATCACTG	TCTTTCCATCCCACTTCTGC
MYF5	AGGGAGCAGGTGGAAAACTAC	AGGAGCTTTTATCCGTGGCAT
MYH1	CCCCATGAACCCTCCCAAAT	GGGTTGACGGTGACACAGAA
MYH3	CGTGGTCGACTCGAAGGAAG	GTCCTCGATCCGGTCAAACT
MYOD	GACGGCACCTATTAAGCGA	CACGATGCTGGACAGACAGT
MYOG	CCAGGAACCCCACTTCTATGAC	GTAGCCTGGTGGCTCAAAGC
PAX7	GGTGGGGTTTTCATCAATGG	GTCTCTTGGTAGCGGCAGAG
PDGFRA	GACTCGAGGTGGGAGTTTCC	TGGCTGTGGGTTTTAGCATCT
PPARG	TTAGATGACAGCGACCTGGC	CACATTCAGCAAACCTGGGC
RPL4	CAAGAGTAACTACAACCTTC	GAACTCTACGATGAATCTTC

### Immunofluorescence

Cells were fixed in 4% paraformaldehyde and permeabilized at room temperature either in 1:1 methanol:acetone solution for 10 min (for MYHC) or in 0.5% Triton‐X‐100 (ThermoFisher Scientific) for 15 min (for PAX7 and MYOD). Subsequently, nonspecific antibody binding was blocked by incubating the samples at room temperature in either protein block solution (Abcam, Cambridge, UK) for 1 h or in homemade blocking solution (10% goat serum, 2% BSA, 0.25% Triton® X-100 in 1× PBS) for 30 min, respectively. Primary antibodies (Table 1) were then incubated for 16 h at 37 °C (PAX7) or 4 °C overnight (MYOD and MYHC) in blocking solution (MYOD and PAX7) or antibody diluent reagent (MYHC; Life Technologies). After washing in PBS or PBS plus 0.05% Triton X (PBST), MDPCs were incubated with secondary AF488-conjugated goat anti-mouse antibody for 1 h. Cells were washed in PBS or PBST for a further 3 times and overlayed with mountant containing DAPI (Sigma‐Aldrich) and subsequently covered with a coverslip. Images were captured on Axiovert 25 and Axiovert 200M inverted microscopes and analysed with Zen Software (Zeiss, Oberkochen, Germany). No antibody (unstained) and secondary antibody only controls were used (Supplementary Fig. 3).

### Cell differentiation

#### Myogenesis

Cells were differentiated as per a previously published protocol^63^ with minor changes. In short, MDPCs (5 × 10^4^) were seeded in triplicate on rh-Laminin 521 (0.5 µg/cm^2^, Life Technologies)-coated 24-well or 96-well plates plates with growth media (DMEM supplemented with 10% FBS, 5 ng/ml bFGF and 1% PS). Once cells reached 70% confluency, media was changed to muscle proliferation media containing 80 nM dexamethasone (Sigma Aldrich), 10% FBS and 1% PS, until they reached full confluence, at which point (Day 0) media was replaced with serum free medium supplemented with 1% Insulin-Transferrin-Selenium (100×, Life Technologies) and 1% PS. Samples were taken on Days 0, 3 and 5 in TRIzol reagent and stored at – 80 °C, or cells on Day 5 were fixed in 4% PFA.

#### Adipogenesis

MDPCs (5 × 10^4^) were seeded in triplicate wells onto 24-well or 96-well plates coated with collagen (50 µg/ml, Sigma Aldrich) in growth media (DMEM HG, 10% FBS, 5 ng/ml bFGF, 1% PS). Once they reached 90% confluence (Day 0), cells were induced to differentiate in the presence of IBMX (0.5 mM, Stemcell Technologies, Cambridge, UK), dexamethasone (1 µM, Sigma Aldrich), indomethacin (100 µM, Stemcell Technologies), Insulin (10 µg/ml, Sigma Aldrich), 10% FBS and 1% PS. After 4 days, they were placed in adipocyte maintenance media (DMEM supplemented with 10% FBS, 10 µg/ml Insulin and 1%PS) until day 14 with media changes every 2–3 days. Samples were taken in TRIzol on Days 0, 7 and 14, and adipocytes were visualised by staining with Oil Red O (ORO, 0.4%, Sigma Aldrich) for 10min at room temperature followed by washing with distilled water. Images were captured in a Zeiss Axiovert 25 inverted microscope.

#### Osteogenesis

Cells were differentiated as previously described^64^. In short, once they reached 90% confluence cells were placed in a mix of high glucose and low glucose DMEM (50:50 v/v; Sigma-Aldrich) supplemented with 10% FBS, 1% Penicillin/Streptomycin, 100 nM dexamethasone (Sigma-Aldrich), 10 mM sodium β-glycerophosphate (Sigma-Aldrich) and 0.1 mM stabilized ascorbic acid (Sigma-Aldrich). After 3 days, cells were switched to DMEM low glucose supplemented as above and cultured for up to 17 days with media change every 3 days. Control cells were maintained in Hams F10 nutrient mix, 10% FBS, and 1% Penicillin/Streptomycin. After diifferentiation cells were fixed in paraformaldehyde (4%) for 15 min and stained with Alizarin Red (2%; pH 4.2) for 30–45 min. Samples were imaged in a Zeiss Axiovert 25 Inverted Phase microscope using Zen Blue software (Advanced Micro Devices).

#### Chondrogenesis

Cells were differentiated using the StemPro Chondrogenesis Differentiation Kit (A1007101, Thermofisher). Briefly, cells were seeded in 10 µl micromasses in a 96 well plate (80,000 cells/each) and kept for 2 h in a humidified chamber in the incubator before differentiation medium was added. After 14 days, the chondrogenic micromasses were fixed in paraformaldehyde (4%) for 15 min and stained for 30–45 min with Alcian Blue (1%; Sigma). Samples were imaged in a Zeiss Axiovert 25 Inverted Phase microscope using Zen Blue software (Advanced Micro Devices).

### Statistical analysis

Statistical analyses were performed using Minitab 20 Statistical Software 2022 (Computer software, Pennsylvania, USA). Unless normally distributed (Kolmogorov–Smirnoff test, P > 0.01), data were log-transformed before analyses using one-way or two-way ANOVA and subsequent pair-wise mean comparisons with Tukey’s or Bonferroni tests, with statistical significance set at P < 0.05, while mean differences with P < 0.1 were taken as approaching signficance. All Graphs were produced using GraphPad PRISM software (version 9.0.2; La Jolla, CA, USA).

### Supplementary Information

Supplementary Information 1.

Supplementary Information 2.

Supplementary Information 3.

## Data Availability

All data is available within the manuscript.
