# Supplementary figures and images for: Derivation and long-term maintenance of porcine skeletal muscle progenitor cells
Source: Sci Rep. 2024 Apr 23;14:9370. doi: 10.1038/s41598-024-59767-0 (PMC11039667; doi:10.1038/s41598-024-59767-0)

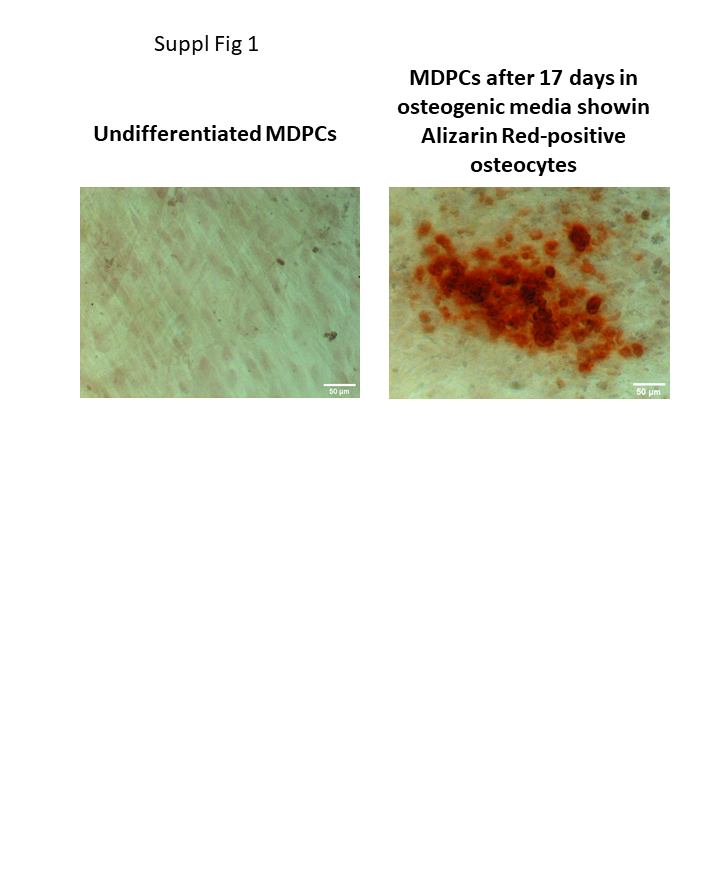

Supplement: Supplementary file 1 — Supplementary Information 1. [file 41598_2024_59767_MOESM1_ESM.tif]

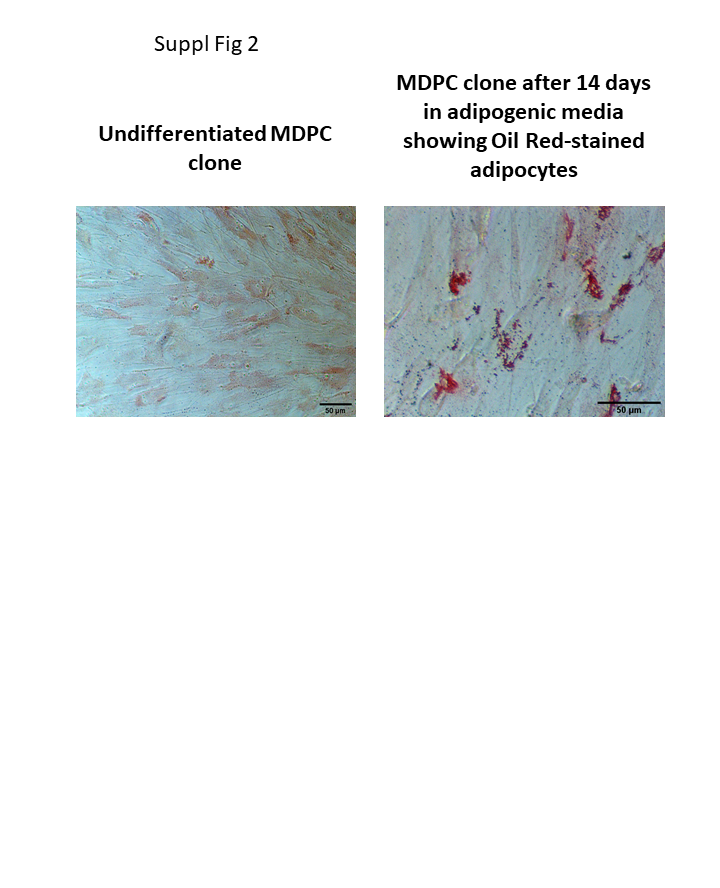

Supplement: Supplementary file 2 — Supplementary Information 2. [file 41598_2024_59767_MOESM2_ESM.tif]

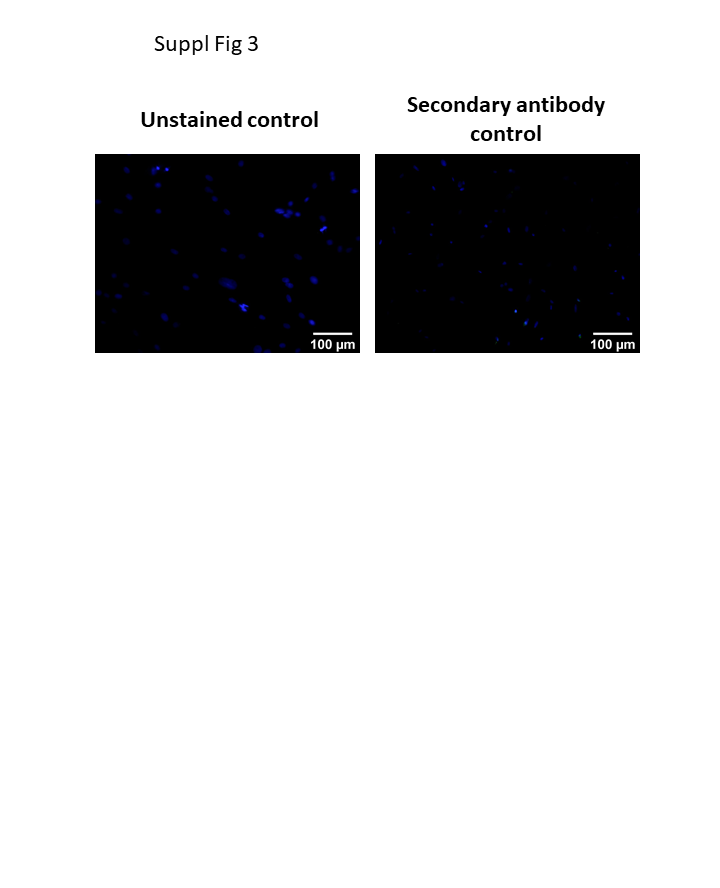

Supplement: Supplementary file 3 — Supplementary Information 3. [file 41598_2024_59767_MOESM3_ESM.tif]
